# Establishing a role for the visual complexity of linguistic stimuli in age-related reading difficulty: Evidence from eye movements during Chinese reading

**DOI:** 10.3758/s13414-019-01836-y

**Published:** 2019-08-13

**Authors:** Lin Li, Sha Li, Fang Xie, Min Chang, Victoria A. McGowan, Jingxin Wang, Kevin B. Paterson

**Affiliations:** 1 Academy of Psychology and Behavior, Tianjin Normal University, Hexi, District, Tianjin, 30037 China; 2 Department of Psychology, Fujian Normal University, Fuzhou, China; 3 Department of Neuroscience, Psychology and Behaviour, University of Leicester, George Davies Centre, University Road, Leicester, LE1 9HN UK

**Keywords:** Aging, Pattern complexity, Eye movements, Chinese reading

## Abstract

Older adults experience greater difficulty compared to young adults during both alphabetic and nonalphabetic reading. However, while this age-related reading difficulty may be attributable to visual and cognitive declines in older adulthood, the underlying causes remain unclear. With the present research, we focused on effects related to the visual complexity of written language. Chinese is ideally suited to investigating such effects, as characters in this logographic writing system can vary substantially in complexity (in terms of their number of strokes, i.e., lines and dashes) while always occupying the same square area of space, so that this complexity is not confounded with word length. Nonreading studies suggests older adults have greater difficulty than young adults when recognizing characters with high compared to low numbers of strokes. The present research used measures of eye movements to investigate adult age differences in these effects during natural reading. Young adult (18–28 years) and older adult (65+ years) participants read sentences that included one of a pair of two-character target words matched for lexical frequency and contextual predictability, but composed of either high-complexity (>9 strokes) or low-complexity (≤7 strokes) characters. Typical patterns of age-related reading difficulty were observed. However, an effect of visual complexity in reading times for words was greater for the older than for the younger adults, due to the older readers experiencing greater difficulty identifying words containing many rather than few strokes. We interpret these findings in terms of the influence of subtle deficits in visual abilities on reading capabilities in older adulthood.

Evidence from eye-movement studies in alphabetic languages (English, German) consistently shows that older adults (aged 65+ years) experience greater difficulty reading compared with young adults (typically ages 18–30 years; for a review, see Gordon, Lowder, & Hoedemaker, 2015). Moreover, older adults tend to have greater difficulty identifying words during reading (Kliegl, Grabner, Rolfs, & Engbert, 2004; McGowan, White, Jordan, & Paterson, 2014; Rayner, Reichle, Stroud, Williams, & Pollatsek, 2006; Rayner, Yang, Schuett, & Slattery, 2013; Whitford & Titone, 2017), which is taken as evidence for slower lexical processing in older age (Laubrock, Kliegl, & Engbert, 2006; Rayner et al., 2006; see also McGowan & Reichle, 2018). Recent extensions to this work additionally show that similar patterns of age-related reading difficulty are observed for nonalphabetic languages like Chinese (S. Li, Li, Wang, McGowan, & Paterson, 2018; J. Wang, Li, Li, Xie, Chang, et al., 2018; J. Wang, Li, Li, Xie, Liversedge, et al., 2018; Zang et al., 2016).

Normative aging effects on reading are widely attributed to subtle visual and cognitive changes in older adulthood (see Gordon et al., 2015), although the relative contribution of these factors is poorly understood. Substantial evidence also shows that older adults suffer more, compared with young adults, when the visual characteristics of text (e.g., font difficulty, removal or reduction of interword or interletter spacing) make word recognition more difficult (see, e.g., S. Li et al., 2019; McGowan et al., 2014; Rayner et al., 2006; Rayner et al., 2013). With the present research, we focused on the influence of the visual complexity of written language, which may make an important perceptual contribution to age-related reading difficulty. This appears to vary across different writing systems and type styles (Pelli, Burns, Farell, & Moore-Page, 2006), but is naturally confounded with word length in alphabetic languages, including English. We therefore investigated effects in Chinese. Text in this writing system is composed of box-like logograms, called characters, which can vary considerably in their number of component strokes (lines, dashes), while always occupying the same square area of space (see Hoosain, 1991, 1992). For instance, simple characters may be created from a single stroke (e.g., 一 [“yi”], meaning “one”) while more complex characters can contain upwards of 20 strokes (such as 罐 [“guan”], meaning “pot”). In these examples, a single character corresponds to a word. However, most words in Chinese contain two (and sometimes more) characters, although spaces are not used to demarcate word boundaries in text. Consequently, while Chinese is uniquely well suited for investigating effects of visual complexity on reading unconfounded by word length (X. Li, Zang, Liversedge, & Pollatsek, 2015; Zang, Liversedge, Bai, & Yan, 2011), it is important to consider effects for both single-character and multicharacter words.

Numerous eye-movement studies show that character complexity, in addition to both lexical frequency and the frequency of usage of individual characters, has an important influence on eye-movement control in Chinese reading (Just & Carpenter, 1987; X. S. Li, Bicknell, Liu, Wei, & Rayner, 2014; Liversedge et al., 2014; Ma & Li, 2015; Yang & McConkie, 1999; Yu, Zhang, Priest, Reichle, & Sheridan, 2018; Zang et al., 2016; see also Yan, Tian, Bai, & Rayner, 2006). For instance, both Just and Carpenter (1987) and Liversedge et al. (2014) found that one-character words containing fewer strokes have shorter reading times, while Liversedge et al. also reported that less complex one-character words are skipped more often (i.e., readers are more likely to move their eyes past the words without fixating them). Ma and Li (2015) in addition reported similar effects of character complexity on reading times for two-character words. Such findings indicate that the visual complexity of characters can influence the time taken to identify a word, and perhaps also the extent to which characters can be preprocessed in parafoveal vision (so that words might be skipped). Research to date has focused on effects for skilled young adults. However, Zang et al. (2016) showed that the complexity of a single-character word has an larger influence on reading times (but not word-skipping rates) for older relative to younger adults, although these effects were complicated by complex interactions between these variables and lexical frequency. Such findings nevertheless suggest that the visual complexity of characters is a specific source of age-related difficulty in Chinese reading. According, the present research investigated this issue further, using a simpler design in which we controlled the lexical frequency of target words to more clearly reveal age differences in effects of visual complexity. In addition, we investigated effects for two-character target words to establish that adult age differences in the effects of character complexity on eye movements during reading extend beyond the recognition of single characters.

Such findings would demonstrate the generality of character complexity effects and add to our understanding of age differences in eye-movement control during Chinese reading. Moreover, the findings are important in the context of evidence from nonreading studies suggesting that character complexity is a source of perceptual difficulty for older adults. For instance, assessments of character legibility show that higher acuity is required to recognize characters with more strokes (Zhang, Zhang, Xue, Liu, & Yu, 2009; Zhang, Zhang, Xue, & Yu, 2007). Therefore, as older adults typically have lower acuity (e.g., Elliott, Yang, & Whitaker, 1995), they may have particular difficulty recognizing more complex characters. Other evidence comes from studies of the visual span for Chinese characters (i.e., how many characters can be recognized on a single glance without moving the eyes). This research typically uses tasks in which linguistic stimuli (e.g., characters) are presented briefly at a central fixation point or locations to the right and left of this point, and span size is calculated as the range of locations across which stimuli can be recognized and reported accurately (for details, see, e.g., Legge et al., 2007). Studies show that span size is smaller for more complex characters (Wang, He, & Legge, 2014). However, this complexity effect is observed only when characters are presented in trigrams, and not individually, suggesting the effect is due to visual crowding (i.e., difficulty recognizing an object, such as a character, when surrounded by visually similar objects; e.g., Bouma, 1970; Whitney & Levi, 2011). Other research using the same task and alphabetic stimuli shows that older adults are more susceptible to visual crowding (Liu, Patel, & Kwon, 2017). Moreover, a recent study using this task with Chinese characters showed span size for complex characters is even smaller for older than for younger adults (Xie et al., 2019), providing further evidence that character complexity is a source of age-related perceptual difficulty. Such findings may be important in relation to accounts that assume Chinese word recognition begins by extracting character stroke information (Taft, Liu, & Zhu, 1999). In particular, if older readers have specific difficulty processing stroke information in visually complex characters, this may impede their recognition of words relative to younger adult readers.

With the present research, we aimed to provide a clear demonstration of adult age differences in the effects of the visual complexity of Chinese characters during reading. To do so, we examined the eye movements of young and older adults while reading sentences that included one of a pair of two-character target words. These were matched carefully for lexical frequency and contextual predictability, but were composed of characters that were either high or low in visual complexity, defined as numbers of character strokes (see Fig. 1). Theoretical accounts predict that older adults experience greater reading difficulty compared with young adults (e.g., Rayner et al., 2006), and we expected to replicate previous demonstrations of this age-related reading difficulty in Chinese (see S. Li et al., 2018; Wang, Li, Li, Xie, Chang, et al., 2018a; Wang, Li, Li, Xie, Liversedge, et al., 2018b; Zang et al., 2016). We also expected to replicate previous findings showing that the visual complexity of Chinese characters has an important influence on eye-movement control in reading, such that words containing more complex characters are skipped less often and have longer reading times (Just & Carpenter, 1987; X. S. Li et al., 2014; Liversedge et al., 2014; Ma & Li, 2015; Yang & McConkie, 1999; Yu et al., 2018; Zang et al., 2016). Crucially, if visual complexity is an important source of perceptual difficulty for older readers, as previous research suggests (Zang et al., 2016), we may observe a larger increase in reading times, and perhaps also a greater reduction in skipping rates, for words containing more complex characters for older compared with younger adult readers.

**Fig. 1:**
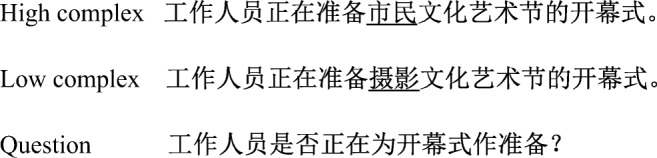
Example sentence and comprehension question. *Note.* High-complexity and low-complexity target words are shown underlined but were presented normally in the experiment. The sentences translates as “The staff are preparing the citizen/photography culture and art festival opening ceremony.” The question translates as “Are the staff preparing the opening ceremony?”

## Method

The study was conducted in accordance with the principles of the Declaration of Helsinki, and all participants gave informed written consent.

### Participants

Participants were 30 young adults ages 19–27 years (*M* = 23 years) and 30 older adults ages 65–88 years (*M* = 73 years) from Tianjin Normal University and the local Tianjin community. All were native Chinese readers with no history of visual or reading impairment (e.g., dyslexia). The two age groups were closely matched for years of formal education (young adults, *M* = 16 years, range: 13–18 years; older adults, *M* = 15 years, range: 11–19 years), *t*(58) = 1.62, *p* = .11, screened for normal visual acuity (better than 20/40 in Snellen values) using a Tumbling E chart (Taylor, 1978), and the older adults were assessed for normal cognitive abilities using the Beijing version of the Montreal Cognitive Assessment (Nasreddine et al., 2005). The young adults had higher acuity (*M* = 20/20, range: 20/16–20/25) than the older adults (*M* = 20/29, range: 20/21–20/35), *t*(58) = 9.77, *p* < .001, as is typical for these age groups (Elliott et al., 1995). We also screened participants for vocabulary knowledge and working/short-term memory capabilities. Normal vocabulary knowledge (i.e., the ability to recognise and define words) was assessed using the WAIS-III Chinese version Vocabulary Knowledge subtest (Wechsler, Chen, & Chen, 2002). Memory capabilities using the WAIS-III Digit Span subtest, which involves repetition of digit sequences of varying length in both the same and reversed directions (Wechsler, 1997). Compared with the young adults, the older adults had slightly lower vocabulary scores (young adults, *M* = 15.9, *SD* = 1.2, range: 14–18; older adults, *M* = 14.5, *SD* = 1.9, range: 10–17), *t*(58) = 3.30, *p* < .01, and smaller digit spans (young adults, *M* = 14.8, *SD* = 2.2, range: 11–18; older adults, *M* = 12.1, *SD* = 2.8, range: 8–19), *t*(58) = 4.12, *p* < .01 (note that values refer to raw test scores, not vocabulary or digit span size).

### Materials and design

Stimuli were 60 sets of sentences, each including one of a pair of interchangeable two-character targets words, in simplified Chinese. Following previous eye-movement research, we defined the visual complexity of characters in terms of number of component strokes (e.g., Liversedge et al., 2014), although this has been shown to correlate well with other methods for assessing visual complexity (see H. Wang et al., 2014). Characters in target words in the present experiment had either more than nine strokes and so of high visual complexity, or seven or fewer strokes and so low visual complexity. Words with high and low complexity characters differed significantly in number of strokes (high complexity, *M* = 22.5, *SD* = 2.8; low complexity, *M* = 10.3, *SD* = 2.1), *t*(59) = 27.43, *p* < .001, as did their first and second characters: first character, high complexity (*M* = 11.3, *SD* = 1.6), low complexity (*M* = 5.1, *SD* = 1.3), *t*(59) = 22.43, *p* < .001; second character, high complexity (*M* = 11.3, *SD* = 2.2), low complexity (*M* = 5.2, *SD* = 1.5), *t*(59) = 18.96, *p* < .001. Lexical frequency, character frequency, and contextual predictability are important determinants of reading times and skipping rates for words in Chinese (e.g., X. S. Li et al., 2014; Yan et al., 2006), and so were controlled as carefully as possible. The high and low complexity words were closely matched for lexical frequency (high complexity = 52.4 counts/million, low complexity = 52.5 counts/million), *t*(59) = .56, *p* = .58, using to the SUBTLEX-CH corpus (Cai & Brysbaert, 2010). However, the frequencies of the first and second characters in words were less well matched: first character: high complexity = 492 counts/million, low complexity = 925 counts/million, *t*(59) = 2.21, *p* < .05; second character: high complexity = 275 counts/million, low complexity = 1,120 counts/million, *t*(59) = 3.41, *p* < .05. We therefore included character frequencies as covariates in key statistical analyses. To assess contextual predictability, we conducted a cloze task with 10 young adult Chinese readers (who did not participant in the experiment). This showed that high and low complexity target words were equally predictable in sentences (high complexity, 3.0% words guessed correctly; low complexity, 4.8% words guessed correctly; *t* < 1). Sentence stimuli were 15–19 characters (*M* = 18) long, and target words were always located near the middle of each sentence.

Sentence frame and target word combinations were divided into two lists, each containing one version of each sentence frame and target word combination and an equal number of sentences containing high or low complexity words. Fifteen participants from each age group were randomly allocated to each list. Accordingly, a mixed experimental design was used with the between-participants factor age group (young adult, older adult) and within-participants factor complexity (high, low). Dependent variables were sentence reading time and eye movement measures.

### Apparatus and procedure

An EyeLink 1000 eye tracker with high spatial resolution (<.01° RMS) recorded right-eye gaze location every millisecond during binocular viewing. Sentences were displayed in Song font as black-on-white text. At 75 cm viewing distance, each character subtended approximately 1° horizontally and of normal size for reading.

Participants were tested individually and instructed to read normally and for comprehension. At the start of the experiment, a three-point horizontal calibration procedure was performed across the same line as each sentence was presented (ensuring .30° or better spatial accuracy for all participants, so accurate to within half a character). Calibration accuracy was checked before each trial, and the eye-tracker recalibrated if accuracy was below this criterion. At the start of each trial, a fixation square equal in size to one character was presented on the left side of the screen. Once the participant fixated this square, a sentence was presented with its first character replacing the square. Participants pressed a response key once they finished reading each sentence. The sentence was then replaced by a yes/no comprehension question on 25% of trials and the participant responded by pressing one of two response keys. The experiment lasted approximately 40 minutes for each participant.

## Results

Accuracy answering comprehension questions (analysed using linear mixed-effects models, see subsequently) was high for all participants and similar for young (*M* = 95%) and older (*M* = 93%) adults (β = .02, *SE* = .02, *z* = 1.03), indicating that both age groups understood the sentences well. Following standard procedures, short (<80 ms) and long (>1,200 ms) fixations were removed. This affected a similar proportion of fixations for the two age groups, 2.2% of fixations for young adults, 2.4% of fixations for the older adults, *t*(58) = .92, *p* = .36. Trials in which there was track loss or error were also excluded (affecting <2% of trials).

Sentence-level and target word-level measures are reported (see Rayner, 2009). Sentence-level measures were informative about age differences in reading behaviour. We examined sentence reading time (time from the onset of a sentence display until the participant pressed a key to indicate they had finished reading), number of fixations, average fixation duration, forward saccade length (mean length, in characters, of forward eye movements) and the number of regressions (backwards eye movements).

Target word-level measures included measures informative about both the first-pass processing and later integration of words. First-pass measures assessed the initial processing of a word prior to a fixation to its right or a regression to its left. The measures used were word skipping (probability of not fixating a word during first-pass), first-fixation duration (duration of the first fixation on a word during first pass), single-fixation duration (duration of the first fixation on a word receiving only one first-pass fixation), re-fixation probability (probability of a word receiving more than one first-pass fixation), gaze duration (sum of all first-pass fixations on a word) and regressions-out (probability of a regression from a word). Total reading time (sum of all fixations on a word) and regressions-in (probability of a regression to the target word) provided measures of later integration.

Data were analysed by linear mixed-effects models (LMMs; Baayen, Davidson, & Bates, 2008) using R (R Development Core Team, 2016) and the lme4 package (Bates, Mächler, Bolker, & Walker, 2014). For binomial variables, generalized LMMs were conducted with the Laplace approximation. A maximal random effects structure was used (Barr, Levy, Scheepers, & Tily, 2013), with participants and items as crossed random effects. For sentence-level measures, age group was a fixed factor, and for target word measures, age group, complexity, and their interaction were fixed factors. Older adults read more slowly than young adults, and so differential effects of visual complexity may reflect a multiplicative slower processing for words composed of more rather than less complex characters by older, relative to younger, adult readers (see, e.g., Faust, Balota, Spieler, & Ferraro, 1999). We adjusted the word-level data to take account of this using log transformation (Loftus, 1978; Wagenmakers, Krypotos, Criss, & Iverson, 2012). Reading-time effects based on both untransformed and log-transformed analyses are reported for transparency and comparability as key previous studies of complexity effects in Chinese reading report effects based on untransformed data (e.g., X. S. Li et al., 2014; Ma & Li, 2015; Yu et al., 2016).

Contrasts of main effects and contrasts to examine interactions were defined using sliding contrasts (the contr. sdif function) in the MASS package (Venables & Ripley, 2002). As there were only two levels of each variable, these produced effect coding for main effects. Following convention, *t/z* > 1.96 were considered significant.

### Sentence-level measures

Table 1 shows sentence-level means and Table 2 summarizes the statistical effects. The analyses show that older adults read more slowly and made more and longer fixations and more regressions than young adults, but with no age group difference in forward saccade length, consistent with evidence from previous Chinese research (S. Li et al., 2018; Wang, Li, Li, Xie, Chang, et al., 2018a; Wang, Li, Li, Xie, Liversedge, et al., 2018b; Zang et al., 2016).

**Table 1:** Means for sentence-level measures

	Young adults	Older adults	AE
Sentence reading time (ms)	3,541 (35)	5,899 (49)	2,358
Number of fixations	13.3 (.12)	20.5 (.21)	7.2
Average fixation duration (ms)	234 (1)	262 (1)	28
Forward saccade length (characters)	2.0 (.01)	2.2 (.02)	0.2
Number of regressions	2.9 (.05)	5.4 (.09)	2.5

*Note.* Standard errors are reported in parentheses. AE = age group effect

**Table 2:** Statistical effects for sentence-level measures

		Sentence reading time	Number of fixations	Average fixation duration	Forward saccade length	Number of regressions
Intercept	β	4,758.00	16.94	248.25	2.10	4.07
	*SE*	168.60	0.63	3.83	0.06	0.23
	*t/z*	28.22	26.94	64.82	35.17	17.52
Age	β	2,429.00	7.19	27.80	0.18	2.61
group	*SE*	320.10	1.20	7.59	0.12	0.45
	*t/z*	7.59*	5.99*	3.66*	1.50	5.86*

*Note.* Asterisks indicate statistically significant fixed-factor effects (*t*/*z* > 1.96)

### Target-word-level analyses

Table 3 shows target-word level means and Table 4 summarizes untransformed and log-transformed statistical effects. Analyses of untransformed analyses produced main effects of age group due to lower skipping rates, higher refixation probabilities, longer reading times, and higher regression rates for the older than the younger adults, consistent with previous research (S. Li et al., 2018; Wang, Li, Li, Xie, Chang, et al., 2018a; Wang, Li, Li, Xie, Liversedge, et al., 2018b; Zang et al., 2016). Main effects of visual complexity in the untransformed data additionally showed that higher complexity words were skipped less frequently, refixated more often and received longer reading times, also in line with previous research (Just & Carpenter, 1987; X. S. Li et al., 2014; Liversedge et al., 2014; Ma & Li, 2015; Yang & McConkie, 1999; Yu et al., 2018; Zang et al., 2016). Analyses of log-transformed reading times produced the same pattern of main effects as the untransformed data.

**Table 3:** Means for word-level measures

	Young adult		Older adult		AE	CE
	High complexity	Low complexity	High complexity	Low complexity		
Word skipping (%)	6.4 (.8)	10.3 (1.0)	3.0 (.6)	4.3 (.7)	−4.7	−2.6
First-fixation duration (ms)	246 (3)	239 (3)	298 (4)	276 (3)	44	14
Single-fixation duration (ms)	248 (4)	236 (4)	292 (5)	275 (4)	41	14
Refixation probability (%)	28.4 (1.6)	19.2 (1.3)	42.0 (1.6)	33.1 (1.6)	13.8	9.1
Gaze duration (ms)	315 (5)	287 (5)	444 (9)	384 (8)	113	44
Regressions–out (%)	8 (.9)	8 (.9)	9 (.9)	10 (1.0)	1.5	−0.9
Total reading time (ms)	426 (10)	372 (8)	672 (14)	630 (15)	251	48
Regressions–in (%)	20 (1.4)	17 (1.3)	30 (1.5)	33 (1.6)	13	−0.3

*Note.* AE = age group effect; CE = complexity effect. Standard errors are shown in parentheses

**Table 4:** Statistical effects for word-level eye-movement measures

		Word skipping	First-fixation duration	Single-fixation duration	Refixation probability	Gaze duration	Regressions–out	Total reading time	Regressions–in
Intercept	β	3.45	264.49	265.26	1	355.12	2.61	521.91	1.22
	*SE*	0.21	5.24	5.36	0.13	11.87	0.13	22.7	0.09
	*t*	16.18	50.48	49.47	7.97	29.92	20.81	22.99	13.94
Intercept	β		5.51	5.52		5.74		6.05	
(log transformed)	*SE*		0.02	0.02		0.03		0.04	
	*t*		286.72	279.5		195.05		161.83	
Age group	β	1.02	45.35	44.06	0.69	112.02	0.23	251.21	0.76
	*SE*	0.38	9.74	9.83	0.24	21.66	0.2	41.43	0.17
	*t*	2.67*	4.65*	4.48*	2.89*	5.17*	1.14	6.06*	4.54*
Age group	β		0.17	0.17		0.28		0.47	
(log transformed)	*SE*		0.04	0.04		0.05		0.07	
	*t*		4.77*	4.51*		5.33*		6.98*	
Complexity	β	0.48	14.36	15.26	0.52	45.39	0.1	50.81	0.03
	*SE*	0.16	4.17	5.01	0.08	8.88	0.12	15.23	0.08
	*t*	3.03*	3.45*	3.05*	6.35*	5.11*	0.82	3.34*	0.4
Complexity	β		0.05	0.06		0.12		0.11	
(log transformed)	*SE*		0.01	0.02		0.01		0.02	
	*t*		3.56*	3.13*		7.97*		4.35*	
Age group	β	0.17	13.58	4.77	0.08	31.41	0.21	10.43	0.43
× Complexity	*SE*	0.32	7.38	8.33	0.16	12.66	0.24	21.5	0.17
	*t*	0.52	1.84	0.57	0.49	2.48*	0.86	0.49	2.57*
Age group	β		0.05	0.02		0.04		0.04	
× Complexity	*SE*		0.02	0.03		0.03		0.03	
(log transformed)	*t*		2.01*	0.56		1.49		1.03	

*Note.* Asterisks to indicate statistically significant fixed-factor effects (*t/z* > 1.96)

Untransformed and log-transformed analyses also revealed an interaction between age group and complexity in early reading time measures (marginal in first-fixation durations and significant in gaze durations for untransformed data, and significant in first-fixation durations for log-transformed data). These were due to a larger complexity effect for the older adults than the younger adults (first-fixation duration, older adults = 22 ms effect, young adults = 7ms effect; gaze duration, older adults = 60 ms effect, young adults = 28 ms effect). These measures are sensitive to early stages of processing associated with the lexical identification of words during reading. Higher character complexity therefore appear to slow word identification more for older, compared with younger, adult readers.

We noted in the Method section that while the lexical frequency of target words was well controlled, the frequencies of first and second characters differed. Both lexical frequency and the frequency of the first character of multicharacter words have been shown to influence reading times (e.g., X. S. Li et al., 2014; Yan et al., 2006). We therefore constructed further LMMs that included first and second character frequency as covariates to assess if these influenced the interaction effects in untransformed gaze durations and log-transformed first-fixation durations. The gaze duration analysis produced a main effect of first character frequency (β = −143.84, *SE* = 39.97, *t* = 3.60), but no main effect of second character frequency (β = 47.65, *SE* = 145.55, *t* = .33), and first character frequency interacted with character complexity (β = 247.49, *SE* = 75.79, *t* = 3.27), due to a larger frequency effect for high-complexity characters. However, there was still an interaction between age group and character complexity (β = 43.26, *SE* = 14.75, *t* = 2.93), and no higher-order interactions. The first-fixation duration analysis showed no effects of first-character or second-character frequency (βs < .1, *t*s < 1.6) but an interaction between age group and complexity (β = 35.03, *SE* = 14.61, *t* = 2.40). Therefore, the key interactions between age group and complexity are not attributable to differences in first or second character frequency.

The absence of an interaction between age group and complexity in word skipping suggests that higher character complexity did not disrupt parafoveal processing more for the older readers. We sought support for this argument using Bayes factors (BFs; Kass & Raftery, 1995) to quantify the relative evidence for additive versus interactive effects of age group and complexity on word skipping. These were computed using the BayesFactor package (see Rouder, Morey, Speckman, & Province, 2012) in R (R Development Core Team, 2016). Marginal likelihood was obtained using Monte Carlo sampling, with iterations set at 100,000 and the scaling factor for *g* priors set to 0.5. Following the interpretation categories specified by Vandekerckhove, Matzke, and Wagenmakers (2014), we interpreted BFs larger than three as weak to moderate support for a model, and BFs larger than 10 as strong support, while BFs less than one provided evidence against a model. The analysis provided weak to moderate support for an additive effects model over an interactive effects model (BF = 5.38). Our findings therefore support an account in which character complexity does not affect parafoveal processing more for older readers.

Finally, the LMM analysis produced an unpredicted interaction in regressions–in, due to a larger complexity effect (more regressions for higher complexity words) for the young adults (older adult effect = −3%, young adult effect = 3%). This showed that the older adults looked back to the more complex words more often. This may be because the older adults spent longer processing these words during first-pass reading and so were less likely to reinspect them compared to the young adults. However, this did not affect reading times for the words and so did not appear to influence integration processes.

## Discussion

Our findings replicate research showing age-related difficulty in Chinese reading (S. Li et al., 2018; Wang, Li, Li, Xie, Chang, et al., 2018a; Wang, Li, Li, Xie, Liversedge, et al., 2018b; Zang et al., 2016). As in these studies, the older adults read more slowly than young adults by skipping words infrequently and making more and longer fixations and more regressions. Our findings also show that more complex words take longer to read and are skipped less often, consistent with other studies that manipulated the visual complexity of words in terms of the numbers of component character strokes (Just & Carpenter, 1987; X. S. Li et al., 2014; Liversedge et al., 2014; Ma & Li, 2015; Yang & McConkie, 1999; Yu et al., 2018; Zang et al., 2016). Our findings are therefore in line with evidence that the visual complexity of characters in words has an important influence on eye movement control in Chinese reading. In particular, reading time effects show that visual complexity influenced how quickly words could be identified, while a word-skipping effect showed this also influenced parafoveal processing. Reading time effects are important in the context of theories that assume Chinese word recognition begins by extracting stroke information (Taft et al., 1999), as such findings confirm the prediction that words that contain more strokes take longer to recognize. Effects of character complexity on word skipping are also relevant to current theories of parafoveal processing in Chinese reading. According to processing-based accounts (e.g., Li, Liu, & Rayner, 2011; Wei, Li, & Pollatsek, 2013), how much information can be processed parafoveally determines saccade targeting, so that longer saccades are made when more characters can be identified parafoveally. The indication from the present findings (and other studies) is that character complexity can influence this process. In particular, the present findings suggest that less complex characters may be easier to identify parafoveally and so skipped more often.

Crucially, the present findings showed a clear age difference in the effects of character complexity on reading times for words. This was due to a higher cost of visual complexity for the older adults, with a larger reading time increase for high compared with low complexity words for older compared with younger adult readers. This effect emerged in reading time measures associated with lexical identification in analyses of both untransformed and log-transformed data, suggesting that older readers have particular difficulty identifying words composed of characters with a greater numbers of strokes. By comparison, the interaction between age group and visual complexity was not significant for word skipping, although as predicted this effect was numerically larger for the young (3.9% effect) than older (1.3% effect) adults. One possibility is that, as older adults skipped the target words infrequently, they may engage in generally more limited parafoveal processing compared with younger readers. As a result, character complexity may only weakly influence their parafoveal processing, which may explain the absence of an age difference in the effects of character complexity on skipping.

Importantly, the present findings are in line with previous research using one-character target words that also showed larger character complexity effects in reading times, but not skipping rates, for older compared with younger adults (Zang et al., 2016). However, these previous findings were complicated by complex interactions with lexical frequency. The present findings therefore help to establish that the visual complexity of Chinese characters is an important source of age-related reading difficulty using a simpler experimental design, while also demonstrating that such effects are obtained for two-character words and so likely to be pervasive in reading. Our findings also resonate with other evidence that older adults suffer more compared with younger adults when the visual characteristics of text make word recognition more difficult (e.g., S. Li et al., 2019; McGowan et al., 2014; Rayner et al., 2006; Rayner et al., 2013). Such difficulties suggest that older adults are more susceptible to perceptual difficulty, most likely due to a combination of subtle visual deficits, including reduced acuity (e.g., Elliot et al., 1995) and increased visual crowding (Liu et al., 2017), which occur naturally in older age (see Owsley, 2011). These deficits may cause older readers to process stroke information more slowly, and so have greater difficulty recognizing words compared to younger readers, in line with accounts emphasizing the importance of stroke information for character recognition (Taft et al., 1999). Moreover, as character complexity is ubiquitous in logographic writing systems, such as Chinese, this may be a pervasive source of perceptual difficulty for older readers of these languages.

In particular, while the present research focused on effects of character complexity during reading for the simplified Chinese script used widely in mainland China, such effects may be even greater for the more traditional script used in Hong Kong and Taiwan, as characters in this script tend to contain more strokes. Whether the same effects can be observed in alphabetic languages is less clear, as there is little variation in the visual complexity of letters, and variation between words is confounded with word length. Letter recognition nevertheless seems to vary across languages and type styles due to differences in visual complexity (Pelli et al., 2006), and so this may be an important consideration if assessing aging effects cross-linguistically or in relation to font difficulty.
